# Temporal regulation of expression of immediate early and second phase transcripts by endothelin-1 in cardiomyocytes

**DOI:** 10.1186/gb-2008-9-2-r32

**Published:** 2008-02-14

**Authors:** Timothy E Cullingford, Thomais Markou, Stephen J Fuller, Alejandro Giraldo, Sampsa Pikkarainen, Georgia Zoumpoulidou, Ali Alsafi, Collins Ekere, Timothy J Kemp, Jayne L Dennis, Laurence Game, Peter H Sugden, Angela Clerk

**Affiliations:** 1 National Heart and Lung Institute Division, Faculty of Medicine, Imperial College London, Armstrong Road, London SW7 2AZ, UK; 2 Clinical Sciences Centre/Imperial College Microarray Centre, Faculty of Medicine, Imperial College London, Hammersmith Hospital Campus, Du Cane Road, London W12 0NN, UK

## Abstract

**Background:**

Endothelin-1 stimulates Gq protein-coupled receptors to promote proliferation in dividing cells or hypertrophy in terminally differentiated cardiomyocytes. In cardiomyocytes, endothelin-1 rapidly (within minutes) stimulates protein kinase signaling, including extracellular-signal regulated kinases 1/2 (ERK1/2; though not ERK5), with phenotypic/physiological changes developing from approximately 12 h. Hypertrophy is associated with changes in mRNA/protein expression, presumably consequent to protein kinase signaling, but the connections between early, transient signaling events and developed hypertrophy are unknown.

**Results:**

Using microarrays, we defined the early transcriptional responses of neonatal rat cardiomyocytes to endothelin-1 over 4 h, differentiating between immediate early gene (IEG) and second phase RNAs with cycloheximide. IEGs exhibited differential temporal and transient regulation, with expression of second phase RNAs within 1 h. Of transcripts upregulated at 30 minutes encoding established proteins, 28 were inhibited >50% by U0126 (which inhibits ERK1/2/5 signaling), with 9 inhibited 25-50%. Expression of only four transcripts was not inhibited. At 1 h, most RNAs (approximately 67%) were equally changed in total and polysomal RNA with approximately 17% of transcripts increased to a greater extent in polysomes. Thus, changes in expression of most protein-coding RNAs should be reflected in protein synthesis. However, approximately 16% of transcripts were essentially excluded from the polysomes, including some protein-coding mRNAs, presumably inefficiently translated.

**Conclusion:**

The phasic, temporal regulation of early transcriptional responses induced by endothelin-1 in cardiomyocytes indicates that, even in terminally differentiated cells, signals are propagated beyond the primary signaling pathways through transcriptional networks leading to phenotypic changes (that is, hypertrophy). Furthermore, ERK1/2 signaling plays a major role in this response.

## Abstract 2

A microarray profiling study of rat cardiomyocytes provides insights into early and second phase transcriptional responses induced by endothelin-1 and shows the importance of ERK1/2 signaling.

## Background

Expression of immediate early genes (IEGs) constitutes the first phase of gene expression in cellular responses to growth stimuli [1]. IEGs are regulated by pre-existing transcription factors that may be pre-bound to gene promoters. Thus, protein synthesis inhibitors (for example, cycloheximide) do not suppress the increases in expression of IEG mRNAs. Expression of IEG RNAs could reflect changes in their rate of transcription and/or mRNA stability. Intracellular signaling pathways activated by growth stimuli regulate both processes through phosphorylation/dephosphorylation of transcription factors or RNA binding proteins. For example, mitogen-activated protein kinases (MAPKs) such as the extracellular signal-regulated kinases 1/2 (ERK1/2) promote phosphorylation of several transcription factors (for example, Elk1) to modulate their activities [2]. Signaling through another of the MAPKs, p38-MAPK, may regulate mRNA stability through the RNA binding protein Zfp36 [3]. MicroRNAs and antisense RNAs also modulate mRNA levels [4,5], and changes in expression of these regulatory RNAs may also be expected to influence mRNA expression. Although alterations in the levels of targets of micro- or antisense RNAs are essentially secondary, transcription of their target mRNAs is regulated by pre-existing transcription factors and does not require protein synthesis, so they remain within the IEG classification.

Most studies of IEGs focus on proliferating cells entering the cell cycle, often in response to growth factors such as epidermal growth factor (EGF) or platelet derived-growth factor (PDGF). The regulation of some IEGs (for example, AP1 transcription factors) in these systems is well characterized. Genome-wide IEG expression patterns are starting to be established using microarrays but, although IEGs are known to exhibit differences in temporal regulation [1], such studies often aim to identify transcripts modulated at a single 'early' time varying between 30 minutes and 4 h [6,7]. Many known IEGs encode transcriptional regulators that presumably promote expression of downstream (second phase) genes [1]. However, the temporal distinctions between IEG and second phase gene expression are not established and, in the absence of these, results from a single sampling time can be difficult to interpret. A recent microarray study demonstrated acute and transient regulation of IEGs in proliferating cells responding to EGF or serum [8] and highlighted negative feedback of IEGs on gene expression, potentially accounting for the transient nature of some responses. However, feedforward transcriptional signaling and the timing of second phase genes were not defined.

Cardiomyocytes (the contractile cells of the heart) are terminally differentiated. They withdraw from the cell cycle soon after birth, and individual cardiomyocytes enlarge during the postnatal period. Adult cardiomyocytes also hypertrophy in order to accommodate any increase in workload (for example, in hypertensive states). Much attention has focused on identifying stimuli that promote cardiomyocyte hypertrophy, and in elucidating the intracellular signaling pathways they activate. Heterotrimeric Gq protein-coupled receptor agonists (for example, endothelin (ET)-1) are particularly implicated in the hypertrophic response [9,10]. These receptors potently and rapidly (maximal stimulation within 5 minutes) activate protein kinase C, Ras and ERK1/2, which are associated with the development of cardiomyocyte hypertrophy [9,11,12]. It is notable that peptide growth factors such as PDGF promote hypertrophy in cardiomyocytes via protein kinase C and the ERK1/2 cascade [13], just as ET-1 promotes proliferation of fibroblasts and other cells that express the ET_A _receptor [14-16]. It seems likely, therefore, that initial events in the hypertrophic response of cardiomyocytes are not dissimilar to those of proliferating cells as they enter the cell cycle.

Although many studies have explored the intracellular signaling pathways associated with cardiomyocyte hypertrophy, the mechanisms whereby they lead to the developed phenotype are poorly understood. Hypertrophy is characterized by morphological and physiological changes (for example, increased size and myofibrillar content) [17], presumably resulting from changes in gene expression. These changes include an increase in expression of established IEGs (c-*jun*, c-*fos*, c-*myc*, *egr1*), recapitulation of a fetal gene program, and changes in expression of genes associated with cardiomyocyte function [18]. Early studies indicated that IEGs are induced rapidly in cardiomyocytes by hypertrophic stimuli [19,20], suggesting that these promote later changes in mRNA expression associated with hypertrophic cells. However, the hypertrophic phenotype is more generally assumed to reflect directly the early transcriptional changes induced by primary signaling events [21]. An alternative explanation is that phasic expression of early response genes propagates the signal, leading to the end-stage transcriptional changes. Here, we have explored the early transcriptional responses of cardiomyocytes to ET-1, before major increases in protein content and morphological changes associated with hypertrophy are detected. We demonstrate temporal and (in many cases) transient phases of mRNA expression consistent with the concept that the signal is propagated through the transcriptional network, leading to the end-stage changes in gene expression associated with hypertrophy.

## Results

### Temporal and transient expression of RNAs induced by ET-1 in cardiomyocytes

**Figure 1:**
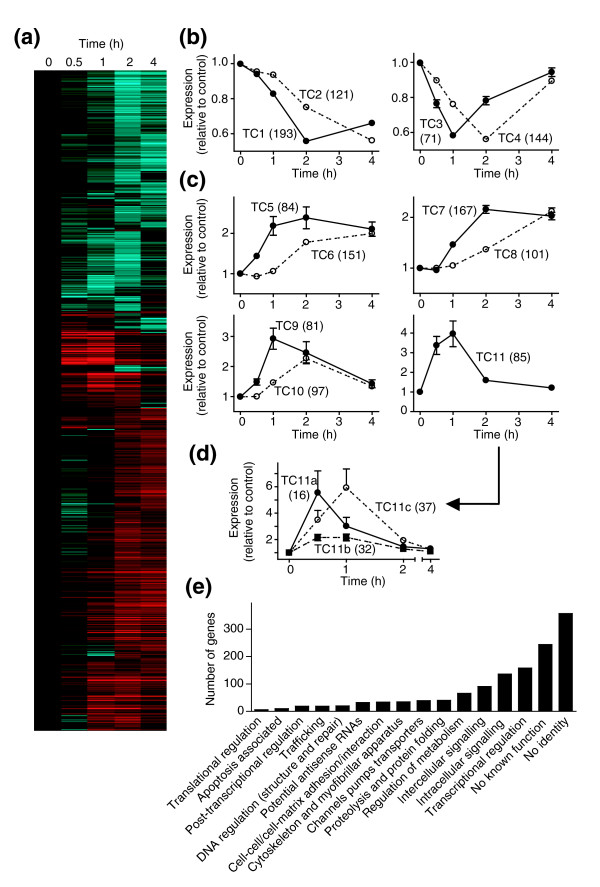
Temporal changes in RNA expression induced by ET-1 in cardiomyocytes. **(a) **The 1,494 probesets identified as significantly (FDR <0.05; 1.5-fold difference) up- or downregulated in response to ET-1 at 0.5, 1, 2 or 4 h were clustered using a Pearson complete correlation and are presented as a heatmap of the mean change at each time (Log_10 _scale: cyan = zero, black = 1, red = 6). **(b,c) **K means clustering generated four groups (TC1-TC4) of downregulated transcripts (b), and seven groups (TC5-TC11) of upregulated transcripts (c). The numbers of different transcripts for each group are shown in parentheses. **(d) **Supervised clustering of TC11 identified three subclusters (TC11a, TC11b and TC11c; numbers of transcripts in each group in parentheses) demonstrating differential temporal and transient expression before 2 h. (Note that three genes exhibited apparent increases in excess of 20-fold (*Egr4*, 50-fold; *c-Fos*, 28-fold; *FosB*, 306-fold) and were excluded from this clustering because of the excessive bias introduced.) Statistical significance (repeated measures one-way ANOVA with Tukey post-test) *p *< 0.05 for all times versus all other times except for the following (not significantly different): TC2, 0.5 versus 1 h; TC3, 0 versus 4 h and 0.5 versus 2 h; TC4, 0.5 versus 4 h; TC5, 1 versus 2 or 4 h, 2 versus 4 h; TC6, 0 versus 0.5 or 1 h; TC7, 0 versus 0.5, 2 versus 4 h; TC8, 0 versus 0.5 or 1 h, 0.5 versus 1 h; TC9, 0 versus 0.5 or 4 h, 0.5 versus 4 h, 1 versus 2 h; TC10, 0 versus 0.5 h, 1 versus 4 h; TC11, 0 versus 2 or 4 h, 0.5 versus 1 h, 2 versus 4 h. (b-d) Results are means ± SEM for the numbers of transcripts given in parentheses. **(e) **Classification of genes regulated by ET-1 according to known or probable function (see Additional data file 1 for details).

Neonatal rat ventricular myocytes [22] were exposed to ET-1 (100 nM, for maximal stimulation [23]) for up to 4 h, and RNA expression profiling was performed with Affymetrix Rat Genome 230 2.0 arrays (>31,000 probesets covering >28,000 well-established genes). Significant changes in expression (false discovery rate (FDR) <0.05; 1.5-fold difference) were identified for 1,306 transcripts (30 minutes, 53 probesets; 1 h, 470 probesets; 2 h, 1,069 probesets; 4 h, 515 probesets; Figure 1a and Additional data file 1). Increases in expression of mRNAs of all but one (*Bcr*) of the 45 established genes that were upregulated at 30 minutes were confirmed by semi-quantitative PCR (SQPCR) or quantitative PCR (QPCR; Additional data file 2). We previously examined the effects of ET-1 on cardiomyocyte RNA expression at the two relatively late time points, 2 and 4 h, using Affymetrix rat genome U34A microarrays. These arrays are not as extensive as the 230 2.0 arrays (approximately 8,000 probesets covering approximately 7,000 established genes), so it was necessary to repeat these times with the higher density arrays for direct comparison with the early times included in this study, but we did identify 77 protein-coding RNAs with significant changes (FDR <0.05; >2-fold difference) in expression. Although the probesets from the U34A and 230 2.0 arrays do not necessarily overlap, 69 mRNAs for well-defined protein-coding genes were identified using either array, in addition to one probeset previously identified as Prss35 but which we have since determined to recognize the antisense strand, and three probesets that may have 'unsafe' annotations and recognize other potential transcripts (Additional data file 3). Linear regression analysis of the changes in expression of these RNAs for the two array studies indicate that the data were comparable (correlation coefficient r = 0.83; slope = 1.06 ± 0.06). Four protein-coding RNAs we previously reported were not identified with the 230 2.0 arrays. One (Kif3c) was consistently called 'absent'. Three (Csrp3, eIF2c2 and Prkr) showed similar changes as with the U34A arrays, but the changes were not significant.

In our previous study [24], a large proportion of the changes in RNA expression induced by ET-1 in cardiomyocytes at 2-4 h required ERK1/2 signaling. Of the mRNAs of the 45 established genes upregulated by ET-1 within 30 minutes (this study), U0126 (a selective inhibitor of ERK1/2 [25]) inhibited the increase in expression of 28 transcripts by >50% and a further 9 transcripts by 25-50% (Additional data file 2). Only four upregulated transcripts were essentially unaffected by U0126. For the remaining four transcripts, the variation in the response to U0126 was too great for adequate assessment. The specificity of 10 μM U0126 for MAPK kinases 1/2/5 (MKK1/2/5, the kinases immediately upstream of ERK1/2/5 and which promote their activation) is high compared with other related kinases [26]. Since ET-1 does not promote significant activation of ERK5 in cardiomyocytes [24], we conclude that ERK1/2 signaling plays a major role in regulating the early changes in RNA expression induced in cardiomyocytes by ET-1.

Unsupervised (K means) clustering identified 11 transcript clusters (temporal clusters TC1-TC11) according to their temporal regulation (four for downregulated transcripts (Figure 1b); seven for upregulated transcripts (Figure 1c)). Clusters TC1, TC2, TC5, TC6, TC7 and TC8 represented RNAs with changes in expression that were sustained at 4 h. However, RNAs in clusters TC3, TC4, TC9, TC10 and TC11 were not only temporally regulated, but the changes in expression of these transcripts were also transient. Thus, expression of TC3/4/9/10 RNAs had almost returned to control levels within 4 h, whereas TC11 transcripts were elevated only at 0.5 and 1 h. TC11 RNAs, as a whole, showed no difference in expression between 0.5 and 1 h, although some RNAs were expressed at substantially higher levels at either time (for example, c-*fos *and *egr4*; Additional data file 1). Further supervised clustering of TC11 transcripts identified subclusters with significantly greater expression at 0.5 h (TC11a) or 1 h (TC11c), or similar expression at both times (TC11b) (Figure 1d). To avoid incorrect annotations [27], the identities of the genes with RNAs that were significantly changed were established by BLAST search of probe sequences. These genes were then classified as far as possible according to function (Figure 1e), but many RNAs encoded hypothetical proteins or proteins of no known function (18.6%), were derived from intronic regions of established genes (6.5%), or could not be assigned to any established gene (20.8%). Affymetrix expression arrays detect numerous potential antisense transcripts [28], and we identified 32 with altered expression in response to ET-1. Of the genes with assigned function, a large number were associated with transcriptional regulation or intercellular/intracellular signaling. This is consistent with the concept that this early phase of mRNA expression is associated with signal propagation, rather than directly establishing the hypertrophic phenotype. The third largest group of genes would be expected to modulate cellular metabolism, presumably also an adaptation required for hypertrophy to develop.

### Identification of IEGs and second phase RNAs

**Figure 2:**
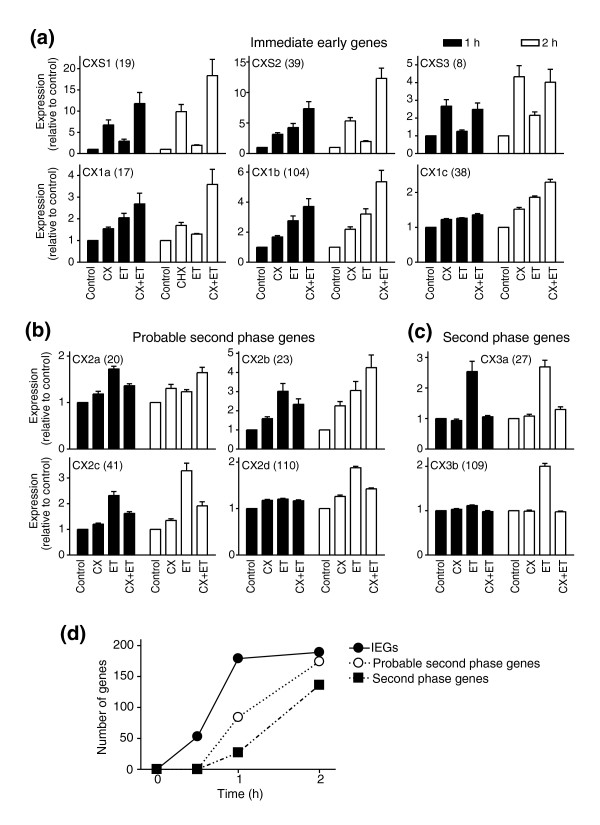
Identification of immediate early and second phase RNAs with increased expression in response to ET-1. Cardiomyocytes were unstimulated (Control), exposed to cycloheximide (CX) alone, or to ET-1 (ET) in the absence or presence of cycloheximide for 1 h (solid bars) or 2 h (open bars). **(a) **RNAs in CXS1, CXS2 and CXS3 (whose induction by ET-1 was further increased by cycloheximide), and CX1a, CX1b and CX1c (whose induction was not inhibited by cycloheximide) were classified as immediate early gene RNAs. **(b) **CX2a, CX2b, CX2c and CX2d RNAs showed partial inhibition of the response to ET-1 at 1 h by cycloheximide and are probably second phase RNAs. **(c) **CX3a and CX3b RNAs were clearly second phase RNAs with >80% inhibition of the response to ET-1 by cycloheximide. The numbers of transcripts in each cluster are shown in parentheses. Statistical significance (repeated measures one-way ANOVA with Tukey post-test) *p *< 0.05 for Control versus ET-1 (2 h; all clusters), Control versus ET-1 (1 h; all clusters except CXS3, CX1c, CX2d and CX3b), Control versus CX (1 or 2 h; all clusters in (a)), ET-1 (2 h) versus CX+ET-1 (2 h) (all clusters except CX2a/b), ET-1 (1 h) versus CX+ET-1 (1 h) (all clusters except CX1b/c, CX2d and CX3b). (a-c) Results are means ± SEM for three independent sets of samples. **(d) **Numbers of IEG versus second phase genes upregulated in cardiomyocytes in response to ET-1 over the first 2 h.

We used cycloheximide to dissect IEGs from second phase transcripts and, because cycloheximide alone affects expression of a number of RNAs (data not shown), we confined our study to RNAs whose expression was increased by ET-1. Expression of mRNAs of established genes upregulated by ET-1 at 30 minutes was not inhibited by 0.02 mM cycloheximide (Additional data file 2). Thus, all can be considered as IEGs (as might be expected). For RNAs upregulated at 1-2 h (589 transcripts), the effects of cycloheximide were studied using microarrays (Additional data file 4). Transcripts were clustered according to time of upregulation by ET-1 and the effect of cycloheximide (Figure 2). Sixty-six transcripts were more potently increased by cycloheximide than by ET-1 (>2-fold at 1 and/or 2 h). These formed 3 subclusters (CXS1, CXS2 and CXS3) with 58 transcripts (CXS1 and CXS2) demonstrating an additive/synergistic response to cycloheximide plus ET-1 (Figure 2a, upper panels). Since these RNAs are increased in the presence of cycloheximide, all are IEGs. Remaining transcripts were clustered according to the degree of inhibition of the ET-1 response by cycloheximide. Clusters CX1a, CX1b and CX1c demonstrated <20% inhibition by cycloheximide and were also classified as IEGs (Figure 2a, lower panels).

Clusters CX2(a-d) were classified as probable second phase RNAs (Figure 2b). Cycloheximide alone slightly increased the expression of RNAs in CX2a, CX2b and CX2c, but partially inhibited (20-80%) the increase in ET-1-induced expression at 1 h, suggesting that full expression of these RNAs (in response to ET-1) is mediated, at least in part, by newly synthesized proteins. For CX2a and CX2b, cycloheximide further increased RNA expression at 2 h. These may operate as IEGs in response to cycloheximide, presumably a consequence of signaling pathways activated by cycloheximide itself (see Discussion). CX2d RNAs were increased only at 2 h and (allowing for the marginal increase by cycloheximide alone) the increase induced by ET-1 was largely inhibited by cycloheximide. CX3a and CX3b RNAs were clearly defined as part of the second phase response, with no effect of cycloheximide alone and >80% inhibition of the ET-1 response (Figure 2c). CX3a therefore represents the earliest second phase RNAs we detected, with increased expression within 1 h. However, 16 (of 27) RNAs did not correspond to any established gene, indicating the relatively poor understanding of this phase of the response. The numbers of IEG RNAs, probable second phase RNAs and clear second phase RNAs that were upregulated by ET-1 are summarized in Figure 2d.

### Differential translation of cardiomyocyte transcripts regulated by ET-1

**Figure 3:**
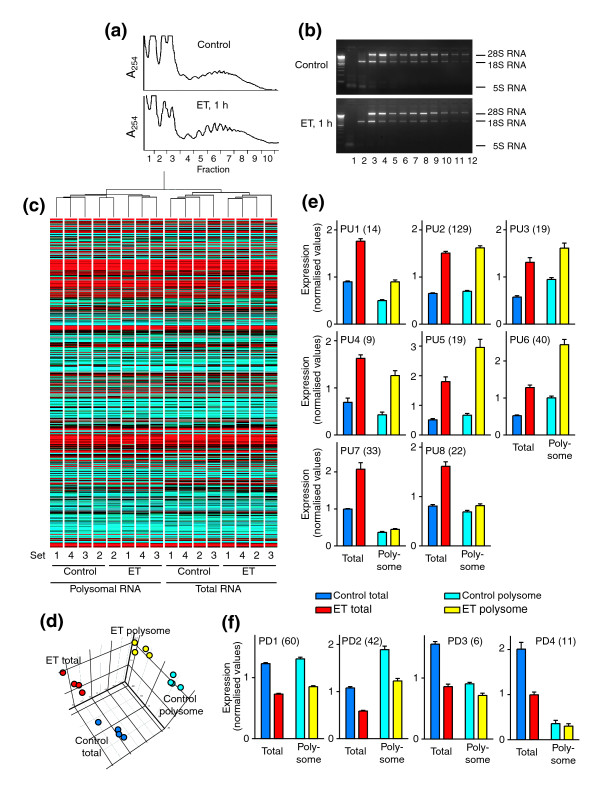
Analysis of cardiomyocyte polysome RNA. Cardiomyocyte extracts from unstimulated cells (Control) or cells exposed to ET-1 (ET) for 1 h were subjected to sucrose density centrifugation. Fraction 1 is the top and fraction 12 the bottom of the gradient. **(a) **A_254 _profiles for sucrose density gradients. **(b) **Agarose gel electrophoresis with ethidium bromide staining of RNA isolated from each fraction highlights 28S, 18S and 5S ribosomal RNA. Fractions 6-11 were pooled for preparation of polysomal RNA for expression profiling. **(c) **All probesets were used for condition clustering (Pearson complete correlation) of polysome and total RNA prepared from individual sets of samples, and a heatmap of the mean normalized expression for each sample is shown (Log_10 _scale: cyan = zero, black = 1, red = 6). **(d) **Principal components analysis identified three components. **(e,f) **RNAs identified as being significantly changed in the total pool in response to ET-1 at 1 h were clustered according to the profiles in total and polysome RNA giving eight groups with increased expression (e) and four groups with decreased expression (f). Results are means ± SEM for four independent sets of samples. Statistical significance (repeated measures one-way ANOVA with Tukey post-test) *p *< 0.05 for Control total versus Control polysome (all clusters except PU2, PU5 and PU8), ET-1 total versus ET-1 polysome (all clusters except PU7, PU8, PD3 and PD4).

There is increasing evidence for translational regulation of selected mRNAs, for example, in response to cellular stresses [29] or as a consequence of Ras signaling [30,31]. To determine whether early transcriptional responses of cardiomyocytes to ET-1 were subject to translational regulation, we compared the expression profiles for total and polysome RNAs (Figure 3a,b) from unstimulated cells and cardiomyocytes exposed to ET-1 (1 h). Total microarray fluorescence values were not significantly different for any of the conditions, but condition clustering separated polysome and total RNA samples (Figure 3c), and principal components analysis identified three principal components for polysome or total RNA isolated from unstimulated or ET-1-treated cardiomyocytes (Figure 3d). Thus, the global profiles are sufficiently similar to permit analysis as a single group, but there are significant differences between each of the conditions.

**Figure 4:**
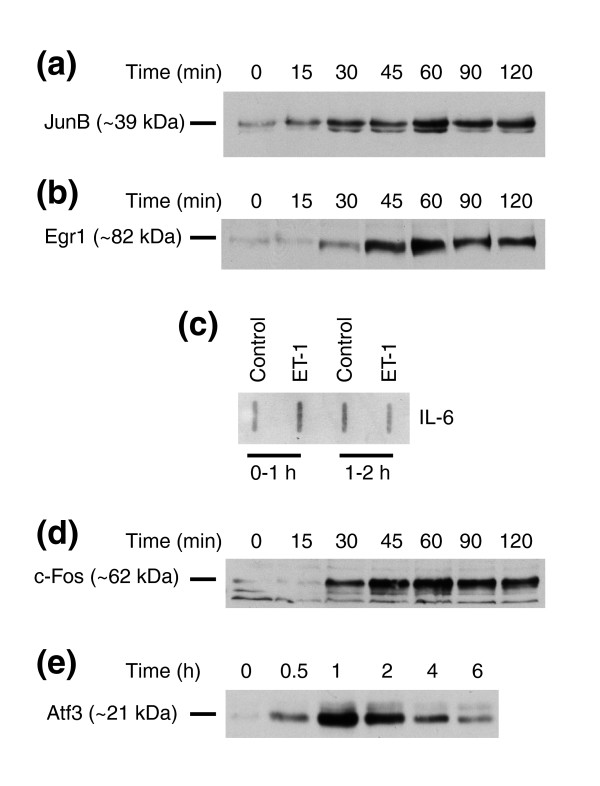
Upregulation of JunB, Egr1, IL-6, c-Fos and Atf3 in cardiomyocytes exposed to ET-1. **(a,b,d,e) **Cardiomyocytes were exposed to ET-1 for the times indicated. Nuclear extracts were analyzed by western blotting using antibodies to JunB (a), Egr1 (b), c-Fos (d) or Atf3 (e). **(c) **Cardiomyocytes were unstimulated (Control) or exposed to ET-1 and the tissue culture medium changed and collected at 1 h (0-1 h secretions) then at 2 h (1-2 h secretions). IL-6 in the media was detected by western blotting using slot blotting to transfer proteins to the nitrocellulose. Experiments were repeated with similar results.

Transcripts with significant changes in expression with ET-1 were clustered according to: increased or decreased expression in response to ET-1; relative expression in polysome or total RNA for unstimulated cells; and relative increase or relative decrease in total and polysome RNA for ET-1-treated cells (Figure 3e,f and Additional data file 5). Approximately 57% of upregulated RNAs (PU1, PU2 and PU3) were increased to the same degree in total and polysome fractions, whether expression levels were relatively higher in total RNA than polysome RNA (PU1), similar in total or polysome RNA (PU2), or lower in total RNA than polysome RNA (PU3) (Figure 3e, upper panels). PU4, PU5 and PU6 transcripts (approximately 24% of upregulated RNAs) were increased to a greater relative extent in polysome RNA (Figure 3e, centre panels), suggesting that they may be preferentially translated. Since PU1-PU6 represent 81% of upregulated transcripts, the majority of upregulated, protein-coding RNAs should be efficiently translated into protein. PU7 and PU8 transcripts were upregulated by ET-1 in the total pool, but were not similarly increased in polysomes (Figure 3e, lower panels). Indeed, PU7 RNAs were largely excluded from the polysomes. These clusters contained a large proportion of RNAs with no association with any protein-coding gene, 11 of which also clustered in CX3a and represent some of the earliest second phase response RNAs (Figure 2c). Approximately 85% of downregulated transcripts (PD1 and PD2) were decreased to a similar degree in total and polysome RNA (Figure 3f). However, small clusters of transcripts were identified that had a substantial decrease in expression in total RNA and a small decrease in polysome RNA and/or were essentially excluded from polysomes in control or ET-1-treated cells (PD3 and PD4). Of the 17 transcripts in these clusters, only two had protein-coding identity. In summary, although there is evidence for preferential recruitment of RNAs to or away from the translational apparatus, for >80% of transcripts with significant changes in expression induced by ET-1, we would expect protein translation to reflect changes in mRNA expression. This has been confirmed by western blotting for upregulated IEGs in PU2 (for example, JunB, Figure 4a), PU3 (for example, Egr1, Figure 4b), PU4 (for example, interleukin (IL)-6, Figure 4c), PU5 (for example, c-Fos, Figure 4d) and PU6 (for example, Atf3, Figure 4e).

### Validation of microarray data

Our microarray data were validated using SQPCR or QPCR for mRNAs of the 45 established genes upregulated at 30 minutes, and QPCR for 22 genes selected from the cycloheximide and polysome clusters. Qualitatively, the differences in expression of all but one of the 30 minute transcripts at 30 minutes (*Bcr*) and one identified at 1-2 h (*Hipk3*) were confirmed (Additional data file 6). This is within our tolerance of FDR <0.05. The apparent fold stimulation observed for arrays versus QPCR often differed but transcripts selected for cluster validation (other than *Hipk3*) showed a high degree of linear correlation across 11 experimental conditions, suggesting that the differences were innate to the experimental approaches. Our validation was generally based on amplification across introns of established genes rather than confined to the probe sequences, and at least some differences (apparent degree of stimulation; failure to validate *Bcr *and *Hipk3*) may be explained by alternative splicing.

## Discussion

The concept of IEGs versus second phase and subsequent phases of gene expression is established in prokaryotic systems and in eukaryotic proliferating cells [1]. However, in more differentiated cell types, this concept has been neglected and it seems to be more generally assumed that classic intracellular signaling events (for example, protein kinase cascades) lead directly to changes in gene expression associated with the end-stage phenotype, such as developed hypertrophy in cardiomyocytes [21]. Even in proliferating cells, knowledge and understanding of IEGs versus second phase genes is rather limited. Here, we have demonstrated that, in cardiomyocytes, ET-1 promotes multiple phases of RNA expression within the first 4 h of stimulation, before major morphological changes or accumulation of total protein are observed (Figure 1). We expected to see the temporal and transient changes in RNA expression over the period studied, but the patterns were perhaps more complex than anticipated. For example, expression of some IEGs was maximal within 30 minutes (cluster TC11a) whereas others were delayed, with maximal expression at 1 h (cluster TC11c). This contrasts with studies of pancreatic cells exposed to glucose and 8-(4-chloro-)phenylthio-cAMP (CPTcAMP) in which IEGs are expressed as long as the stimulus is present, and in which IEGs were dissected from second phase transcripts at 4 h [7]. In cardiomyocytes, expression of IEGs had largely ceased by 4 h. The differences presumably reflect persistent activation of intracellular signaling pathways by CPTcAMP versus activation of endogenous second messengers by endogenous receptors, with transient activation of signaling pathways [10].

Our study was based on stimulation of neonatal rat ventricular myocytes by ET-1. U0126 (an inhibitor or ERK1/2 signaling) attenuated the changes in expression for the majority of the RNAs upregulated in cardiomyocytes by ET-1 at either 30 minutes (Additional data file 2) or 2 h [24], so many of the same transcriptional changes should be detected in other cells responding to agonists that potently activate the ERK1/2 cascade. Indeed, changes in RNA expression reported for other such systems (for example, fibroblasts exposed to PDGF [32] or HeLa cells exposed to EGF [8]) overlap substantially with those identified here. Of the genes with known or probable function, very few were associated with end-stage cardiac hypertrophy [17]. Even in terms of functional groups, these early changes differ markedly from later phases of hypertrophy or heart failure in which genes associated with energy metabolism, extracellular matrix and/or contractility predominate [33-36]. Instead, many were associated with gene or protein expression, or with signaling. These presumably are required for signal propagation towards the end-stage phenotype, and would modulate cardiomyocyte responsiveness to the immediate environment in the intervening period.

Cycloheximide is classically used to define IEGs. However, it also stimulates protein kinase signaling (for example, c-Jun N-terminal kinases (JNKs) and p38-MAPKs [37,38]) and independently promotes expression of certain genes. Consistent with this, we detected significant changes in expression (FDR <0.05; >1.5-fold difference relative to controls) of 1,046 probesets in cardiomyocytes exposed to cycloheximide alone for 70 or 130 minutes (to include the 10 minute pre-incubation period used in association with ET-1), many of which were not changed in response to ET-1 (data not shown). These would seem to be potential IEGs (that is, genes that may be regulated as IEGs in response to other stimuli). For example, cycloheximide increased expression of Gdf15 (3.44 ± 0.36-fold at 70 minutes; 5.69 ± 0.62-fold at 130 minutes), a gene whose expression is increased in cardiomyocytes by H_2_O_2 _[39] but not ET-1, and which may play a role during the development of cardiac pathologies [40,41]. Nevertheless, by confining our analysis here to only RNAs with increased expression in response to ET-1, we could identify IEGs, and inhibition of responses by cycloheximide clearly defined downstream gene expression. We expected to find a number of second phase transcripts after 2 h of stimulation with ET-1, but there were approximately twice as many probable or clear second phase transcripts as IEGs at this time (Figure 2b-d). We also identified a small group of second phase RNAs with increased expression at 1 h, at least some of which are protein-coding (CX3a; Figure 2c and Additional data file 4). Thus, the second phase of RNA expression is induced rapidly in cardiomyocytes and it cannot be assumed that all transcripts that are upregulated at 1-2 h are IEGs, nor can it be assumed that RNAs that are upregulated from 2 h are necessarily part of the second phase response.

Of the IEGs, some (for example, CX1a RNAs) were upregulated by ET-1 at 1 h with reduced expression at 2 h, but were superinduced by cycloheximide at 2 h. For these, synthesis of a negative regulator could be required to suppress the response. One such protein is likely to be Atf3, a negative regulator of gene expression in other systems [42] whose mRNA and protein is increased within 30 minutes (Additional data file 2; Figure 4e). The delayed expression of some IEGs at 2 h (Figure 2a, CX1c and CXS3) relative to others induced at 0.5-1 h (Additional data file 2; Figure 2a, CXS1 and CX1a) may relate to differential effects on mRNA stability and transcription. Although unexplored in cardiomyocytes, a rapid increase in mRNA stabilization increases RNA expression in other systems [43]. Alternatively (or additionally), temporal differences in RNA expression could reflect temporal differences in activation of intracellular signaling pathways that regulate transcription factor activity (for example, stimulation of JNKs by ET-1 in cardiomyocytes is delayed relative to ERK1/2 [44,45]).

Studies of global transcriptional profiles raise the question of whether the mRNAs are translated into protein. Although proteomics studies can address this, methodologies using two-dimensional gel electrophoresis to separate proteins have restrictions with respect to resolution and/or detection levels, whereas analysis of the full proteome by mass spectrometry is highly complex. Furthermore, such studies examine the total level of proteins, favoring those that are more abundant. In cardiomyocytes, we have found it difficult to identify, for example, transcription factors that are expressed at low abundance relative to the myofibrillar or cytoskeletal apparatus using this methodology. In a different approach, other studies have demonstrated selective recruitment of transcripts to polysomes and suggested that translational regulation is an important component of responses to cellular stress or, for example, Ras signaling [29-31]. We detected differential recruitment of transcripts to polysomes in cardiomyocytes, both in the unstimulated state and following exposure to ET-1 (Figure 3), consistent with studies in other systems [29-31]. However, 67% of the transcripts that were changed in cardiomyocytes in response to ET-1 exhibited similar changes in expression in total RNA and polysome RNA, with a further 17% of upregulated transcripts being increased to a greater extent in polysome RNA than in total RNA. Thus, for the majority of transcripts, we expect protein translation to reflect the changes in mRNA abundance. This is the case for the mRNAs/proteins we have studied previously (for example, c-Jun [46], connective tissue growth factor [47]) and those we studied as a consequence of our microarray data, including JunB, Egr1, IL-6, c-Fos and Atf3 (Figure 4).

A principal difference between our study of polysomal RNAs and those of others is that we studied the early phases of a growth response, confining the analysis to RNAs with altered expression in the global RNA pool. Others have examined polysomal RNA specifically in the context of cellular stresses or sustained activation of signaling pathways and did not confine the study only to transcripts that were altered in the global pool [29-31]. However, even in our study, up to 18% of RNAs were preferentially excluded from polysomes (PU7, PU8, PD3 and PD4). These clusters contained a high proportion of RNAs that were not associated with any known protein-coding gene. Although such genes may still be identified for these transcripts, 31 of the total 72 different transcripts corresponded to intronic regions of protein-coding genes. This contrasts with only 7 of 332 transcripts in all other clusters. It remains to be determined whether they are simply by-products generated from splicing of the primary transcripts or if they are themselves functional transcripts. More generally, particularly as we failed to assign any clear function to almost 50% of the RNAs associated with the early response to ET-1 (allowing for transcripts corresponding to hypothetical proteins, transcripts outside any established or predicted gene, transcripts derived from intronic regions and antisense sequences), much remains to be learnt about how the early transcriptional response of cardiomyocytes is integrated and leads to changes in cell function.

## Conclusion

Although it is assumed that protein kinase/phosphatase signaling regulates gene expression in cardiomyocytes and these events are associated with phenotypic changes, the interconnections between the rapid and transient regulation of protein kinases/phosphatases and (with respect to ET-1) hypertrophy have not been established. We have demonstrated that, even though cardiomyocytes are terminally differentiated, a stimulus such as ET-1 still promotes multiple phases of RNA expression, prior to those associated with developed hypertrophy, but which presumably are required for the growth response to develop. Thus, the signal is propagated beyond the primary signaling pathways through transcriptional networks. In cardiomyocytes exposed to ET-1, a large pool of mRNAs were regulated as IEGs over the first 2 h of stimulation, with changes in expression of second phase RNAs from within the first hour. Since ET-1 potently activates ERK1/2 signaling and this pathway plays a major role in regulating RNA expression in this context, we suggest that many of the same genes will be regulated as IEGs or second phase genes in other systems that signal predominantly through the ERK1/2 cascade. Finally, our data on RNA recruitment to cardiomyocyte polysomes indicate that, for most transcripts, translation of IEGs into protein should reflect the changes in mRNA expression. Nevertheless, some transcripts are preferentially excluded from the polysomes and are presumably subject to translational regulation. Overall, our study illustrates the complexities of the early transcriptional response to a single stimulus and contributes to our understanding of the mechanisms associated with signal propagation towards the ultimate and distant phenotypic response.

## Materials and methods

### Cardiomyocyte cultures

Ventricular myocytes from neonatal rats are terminally differentiated [22], but can be maintained in primary culture as a confluent monolayer of spontaneously beating cells. Cardiomyocytes were dissociated from neonatal (1-3 day) Sprague-Dawley rat hearts (at least 4 litters per preparation) and cultured at confluence (4 × 10^6 ^cells per 60 mm Primaria dish) as described previously [39]. Myocytes were plated in 15% (v/v) fetal calf serum (18 h) and then serum-starved (24 h) before being left unstimulated, exposed to 100 nM ET-1 (Bachem (UK) Ltd., St Helens, UK) or 20 μM cycloheximide, or exposed to ET-1 following pre-treatment (10 minutes) with 20 μM cycloheximide.

### Preparation of cardiomyocyte polysomes

Sucrose density gradients were prepared by layering 900 μl each of 1.6 M, 1.4 M, 1.2 M, 1.0 M, and 0.8 M sucrose in buffer A (50 mM Tris-HCl pH 7.4, 100 mM KCl, 2 mM MgCl_2_, 0.1 mg/ml cycloheximide) in 5 ml ultracentrifuge tubes. Gradients were equilibrated overnight (4°C). Cardiomyocytes (16 × 10^6 ^cells per sample) were treated with cycloheximide before harvesting (0.1 mg/ml, 5 minutes), then washed 3 times in ice-cold phosphate-buffered saline containing 0.1 mg/ml cycloheximide. Cells were scraped into 0.3 ml buffer A containing 0.5% (v/v) Triton X100, 0.5% (v/v) Nonidet P40, 0.25 mM dithiothreitol, 1 mg/ml heparin, 40 U/ml RNaseOut (Invitrogen Ltd., Paisley, UK), 0.3 mM phenylmethylsulphonyl fluoride, 0.2 mM leupeptin, 0.002 mM microcystin LR and 0.01 mM trans-epoxy-succinyl-L-leucylamido-(4-guanidino)-butane, and incubated on ice (10 minutes). Extracts were centrifuged (4°C, 20 minutes, 20,000 × g) and the supernatants layered onto the sucrose density gradients. Samples were centrifuged (Sorvall M120SE ultracentrifuge), 4°C, 2 h, 105,000 × g). Fractions (12 in total) were collected by upward displacement whilst monitoring absorbance at 254 nm.

### RNA preparation and microarray hybridization

Total RNA was prepared (4 × 10^6 ^cells per sample) as previously described [48]. Polysome RNA was extracted from fractions 6-11 using RNA Bee (according to the manufacturer's instructions, AMS Biotechnology (Europe) Ltd., Abingdon, UK) and resuspended in 15 μl water. Pooled fractions were incubated with 90 μl 3 M sodium acetate and 180 μl ethanol (5 minutes, 4°C), then centrifuged (4°C, 15 minutes, 20,000 × g). The pellets were washed in 80% (v/v) ethanol and resuspended in 15 μl water. RNA concentrations were determined at A_260_. A_260_/A_280 _ratios were 1.9-2.0. For total or polysome RNA, each sample was generated by combining equal amounts of RNA from three independent preparations of myocytes. Separate samples were generated for hybridization to individual microarrays: n = 3 (prepared from 9 myocyte preparations) for the ET-1 time course (0, 0.5, 1, 2 and 4 h); n = 3 (prepared from 9 myocyte preparations) for control, cycloheximide, ET-1 or cycloheximide/ET-1 (1 and 2 h); n = 4 (prepared from 12 myocyte preparations) for total versus polysome RNA for control or ET-1 (1 h). By using all data for each individual time point, we obtained n = 3 for ET-1 at 0.5 or 4 h, n = 8 for ET-1 at 1 h and n = 6 for ET-1 at 2 h. Unstimulated controls were prepared and hybridized simultaneously with each set of samples. Samples were further purified and concentrated using the RNeasy Minielute Cleanup kit (Qiagen Ltd., Crawley, UK). cDNA and cRNA were synthesized as previously described [48]. Fragmentation of antisense cRNA and hybridization to Affymetrix Rat Genome 230 2.0 arrays were performed at the CSC/MRC Microarray Centre according to their protocol [49]. Data were exported to ArrayExpress (E-MIMR-3, E-MIMR-681).

### Data analysis

Preliminary analysis of hybridization data used Affymetrix GeneChip Operating System, GCOS. The data were imported into GeneSpring GX 7.3.1 (Agilent Technologies UK Ltd., Stockport, UK) as tab-delimited text files. Log_10 _values were used for subsequent analysis with values set to a minimum of 0.01. For each dataset, the data were normalized per array (to the 50th percentile) and then per gene. For studies of the temporal changes in RNA expression induced by ET-1 and the effects of cycloheximide, normalization per gene was to the corresponding controls (prepared and hybridized simultaneously). For the study of expression in total and polysome RNA, data were normalized to the median for each gene for all samples in the experiment (that is, n = 16). The error model was based on deviation from 1 (assumes that most transcripts in the array will not change). A confidence filter was applied and genes were selected if present or marginal in all controls or all of any given time. One-way non-parametric *t*-tests were performed for each transcript for each time relative to the appropriate controls. The FDR was set to <0.05 and multiple testing correction performed using the Benjamini and Hochberg FDR algorithm. Transcripts were filtered on the basis of >1.5-fold difference. All gene identities were confirmed by BLAST search of the probeset sequences using the Entrez nucleotide database [50]. Further BLAST searches for unassigned sequences were performed against the rat genome [51] and, since the rat genome is still less well annotated, the mouse genome [52] (cross-species megaBLAST). Annotations were correct as of August 2007. Genes were classified as far as possible using Gene Ontology classifications associated with rat, mouse and human orthologues [53], taking into account both probable 'function' and 'process'. For genes with conflicting potential functions, further searches were performed using PubMed to ascertain probable biochemical function. Genes were grouped according to their biochemical function in the cell.

For the cycloheximide study, data (n = 3) were obtained for cardiomyocytes treated with cycloheximide (70 or 130 minutes), ET-1 (60 or 120 minutes) or cycloheximide and ET-1 (10 minutes pretreatment with cycloheximide before adding ET-1 for 60 or 120 minutes). Genes were selected for analysis if significantly upregulated in response to ET-1 at 1 or 2 h according to the time course study. Normalized data were exported for supervised clustering using Microsoft Excel. For the study of polysome RNAs, data were obtained for total RNA or polysome RNA from unstimulated cardiomyocytes or cardiomyocytes exposed to ET-1 for 1 h (n = 4, total and polysome RNAs from the same cardiomyocyte preparations). Principal components analysis and condition clustering were performed using GeneSpring GX 7.3.1. Genes were selected for analysis if significantly upregulated or downregulated in response to ET-1 at 1 h according to the time course study. Normalized data were exported for supervised clustering with Microsoft Excel.

K means clustering was performed using GeneSpring GX 7.3.1 using a Pearson correlation allowing up to 100 iterations with testing of 5 additional random clusters. For the time course data (Additional data file 1), 11 clusters proved to be optimum, with clustering converging after 19 iterations. To confirm statistical significance for the cluster sets, normalized values were exported and analyzed by GraphPad Prism 4 (GraphPad Software Inc., San Diego, CA, USA) using repeated measures one-way ANOVA with Tukey post-test. Linear regression analysis was performed using GraphPad Prism 4.

### Reverse transcription-PCR

Primers for SQPCR or QPCR were designed for established genes using published rat sequences (Additional data file 7). Where possible, these were designed across an intron. cDNA was prepared by reverse transcription of RNA samples and SQPCR was performed essentially as described [48] using (for all primer pairs) an amplification cycle of 95°C for 30 s, 59°C for 30 s and 72°C for 30 s. The optimum cycle number determined for each amplicon. Amplified products were visualized on 2% (w/v) agarose gels with Sybr-Safe (Invitrogen) staining and the bands were recorded under UV illumination. The expression of the housekeeping gene glyceraldehyde 3-phosphate dehydrogenase (Gapdh) was monitored in parallel. Products were identified according to size. Bands were quantified by scanning densitometry (ImageMaster 1D, GE Healthcare, Bio-Sciences, Chalfont St Giles, UK) and values were expressed relative to that of Gapdh amplified from the same cDNA sample. QPCR was performed using a 7500 Real-Time PCR System (Applied Biosystems, Warrington, UK). A master-mix containing (per reaction) 12.5 μl Sybr-Green Jump Start Taq Readymix (Sigma-Aldrich Co. Ltd., Gillingham, UK) and 5 μl oligonucleotides (5 pmol each of forward and reverse primers) was aliquoted into Optical 96-well reaction-plates (Applied Biosystems), and cDNA template added (7.5 μl, 1/15 dilution in water). PCR conditions for all primer pairs were 50°C for 2 minutes, 95°C for 10 minutes, followed by 40 cycles of 95°C for 15 s and 59°C for 60 s. Following QPCR, dissociation curve analysis was performed to check for aberrant amplification products. QPCR analysis of Gapdh was performed in each 96-well plate as an endogenous control and the relative quantification protocol was used.

### Western blotting

Western blotting of cardiomyocyte nuclear extracts was performed essentially as described [54] using primary antibodies to JunB (Santa Cruz Biotechnology Inc., distributed by AutogenBioclear, Calne, UK, JunB(N-17)X sc-46X, 1/2,000 dilution), Egr1 (Santa Cruz Biotechnology Inc., Egr1(C-19) sc-189, 1/500 dilution), c-Fos (Santa Cruz Biotechnology Inc., c-Fos (4)X, sc-52X, 1/10,000 dilution) or ATF3 (Santa Cruz Biotechnology Inc., ATF2(C19) sc-188, 1/500 dilution). For IL-6, tissue culture media were collected from unstimulated cardiomyocytes or from cardiomyocytes exposed to ET-1 over 0-1 h, the medium was replaced and then collected again at 2 h. Proteins in 300 μl were transferred to nitrocellulose by slot blotting. Blots were probed with primary antibodies to IL-6 (Santa Cruz Biotechnology Inc., IL-6(R-19) sc-1266, 1/2,000 dilution) and developed as described in [54].

## Abbreviations

CPT-cAMP, 8-(4-chloro-)phenylthio-cAMP; CX, cycloheximide; EGF, epidermal growth factor; ERK, extracellular signal-regulated kinase; ET, endothelin; FDR, false discovery rate; Gapdh, glyceraldehyde 3-phosphate dehydrogenase; IEG, immediate early gene; IL, interleukin; JNK, c-Jun N-terminal kinase; MAPK, mitogen-activated protein kinase; PDGF, platelet-derived growth factor; QPCR, quantitative PCR; SQPCR, semi-quantitative PCR; TC, temporal cluster.

## Authors' contributions

TEC prepared most of the samples for microarrays, performed data validation by QPCR/SQPCR, contributed in the development of the study and coordinated all other experimental aspects. TM prepared the cardiomyocyte polysomes and contributed to the data validation by SQPCR/QPCR. SJF, AG, SP and GZ contributed to the data validation by SQPCR. AG, CE and AA generated the western blot data. TJK prepared some samples for microarrays. JLD and LG were responsible for microarray hybridization and analysis by GCOS. PHS provided helpful discussion and contributed to the writing of the manuscript. AC conceived the research project, performed the GeneSpring and cluster analysis and assembled the manuscript.

## Additional data files

The following additional data are available. Additional data file 1 is a spreadsheet listing the variably expressed genes classified according to function and clustered according to time. Additional data file 2 contains tables listing SQPCR and QPCR validation data for the RNAs that were upregulated by ET-1 at 30 minutes, and the effects of **(a) **cycloheximide or **(b) **U0126. Additional data file 3 is a spreadsheet comparing the data we previously reported for the changes in RNA expression induced by ET-1 at 2 or 4 h [24] with the data generated in this study. Additional data file 4 is a spreadsheet listing the RNAs that were upregulated by ET-1 at 1 or 2 h, clustered according to the effects of cycloheximide. Additional data file 5 is a spreadsheet listing the RNAs that were variably expressed at 1 h, clustered according to their distribution in polysome or total RNA pools. Additional data file 6 is a table of QPCR data for validation of cycloheximide and polysome clusters. Additional data file 7 is a table with details of the primers used for SQPCR or QPCR.

## Supplementary Material

Additional data file 1 Variably expressed genes classified according to function and clustered according to time. Click here for file

Additional data file 2 SQPCR and QPCR validation data for the RNAs that were upregulated by ET-1 at 30 minutes, and the effects of **(a) **cycloheximide or **(b) **U0126. Click here for file

Additional data file 3 Data previously reported for the changes in RNA expression induced by ET-1 at 2 or 4 h are from [24]. Click here for file

Additional data file 4 RNAs that were upregulated by ET-1 at 1 or 2 h, clustered according to the effects of cycloheximide. Click here for file

Additional data file 5 RNAs that were variably expressed at 1 h, clustered according to their distribution in polysome or total RNA pools. Click here for file

Additional data file 6 QPCR data for validation of cycloheximide and polysome clusters. Click here for file

Additional data file 7 Primers used for SQPCR or QPCR. Click here for file
