# Supplementary material for: Temporal regulation of expression of immediate early and second phase transcripts by endothelin-1 in cardiomyocytes
Source: Genome Biol. 2008 Feb 14;9(2):R32. doi: 10.1186/gb-2008-9-2-r32 (PMC2374717; doi:10.1186/gb-2008-9-2-r32)
Supplement: Additional data file 2 — SQPCR and QPCR validation data for the RNAs that were upregulated by ET-1 at 30 minutes, and the effects of (a) cycloheximide or (b) U0126. [file gb-2008-9-2-r32-S2.doc]

**Additional data file 2**

**(a) SQPCR and QPCR analysis of mRNAs upregulated by ET-1 at 30 min.** Cardiomyocytes were unstimulated or exposed to cycloheximide (CX, 10 min) then either continued untreated or exposed to ET-1 (30 min). Established genes with significantly increased expression at 30 min according to microarray analysis were further studied by SQPCR or QPCR. Data are given as fold stimulation relative to unstimulated controls (mean ± SEM, n=4). Increased expression of all genes except Bcr were confirmed.

| **Gene symbol** | **Array data** | **SQPCR** | | | **QPCR** |
| --- | --- | --- | --- | --- | --- |
|  |  | **ET** | **CX** | **CX+ET** |  |
| Arc | 7.39 | 164.50 | 12.28 | 141.68 | --- |
| Atf3 | 3.95 | 16.00 | 3.37 | 17.52 | --- |
| Axud1 | 3.05 | 3.73 | 2.76 | 5.47 | 14.03 |
| Bcr | 1.98 | 0.92 | 1.09 | 0.97 | 1.42 |
| Btg2 | 4.67 | 6.90 | 3.29 | 7.06 | --- |
| Ch25h | 8.52 | 9.84 | 3.79 | 10.58 | 30.98 |
| C8orf4 | 5.41 | 6.59 | 3.08 | 9.53 | --- |
| Cited2 | 2.01 | 2.09 | 1.62 | 2.47 | 6.51 |
| Cyr61 | 4.16 | 2.95 | 1.84 | 2.55 | 11.07 |
| Dusp1 | 3.99 | 7.39 | 4.51 | 11.26 | 15.06 |
| Dusp5 | 2.49 | 3.59 | 1.95 | 5.41 | 3.56 |
| Egr1 | 4.59 | 4.39 | 3.30 | 4.47 | 9.83 |
| Egr2 | 12.65 | 19.10 | 5.61 | 24.62 | 37.73 |
| Egr3 | 21.26 | >999 | 141.37 | >999 | 100.66 |
| Ereg | 1.99 | 3.31 | 3.78 | 5.52 | 7.53 |
| Fos | 27.90 | 34.75 | 11.44 | 40.69 | 175.25 |
| FosB | 306.60 | >999 | 22.28 | >999 | >999 |
| Has2 | 2.50 | 2.99 | 2.18 | 3.64 | 5.74 |
| Ier2 | 5.29 | 2.50 | 2.30 | 2.51 | 12.10 |
| Ier3 | 1.89 | 1.82 | 2.38 | 2.09 | 3.37 |
| Il6 | 4.62 | 11.46 | 13.99 | 16.95 | 15.20 |
| Jun | 2.81 | 2.85 | 2.53 | 3.45 | 10.80 |
| Junb | 4.14 | 2.01 | 1.79 | 2.10 | 37.73 |
| Klf2 | 3.50 | 8.72 | 3.83 | 15.40 | 12.68 |
| Klf4 | 1.98 | 2.48 | 1.50 | 2.61 | 7.16 |
| Klf6 | 2.36 | 2.35 | 1.83 | 2.48 | 5.11 |
| Lif | 3.76 | 5.44 | 4.85 | 9.67 | --- |
| Mat2a | 1.81 | 1.65 | 1.48 | 1.90 | 2.64 |
| Myc | 1.88 | 1.68 | 1.54 | 1.65 | --- |
| Nfil3 | 2.21 | 1.76 | 1.56 | 2.14 | 2.73 |
| Nfkbiz | 2.27 | 3.06 | 3.19 | 4.91 | 4.47 |
| Nr4a1 | 13.57 | 13.42 | 6.64 | 12.31 | --- |
| Nr4a2 | 3.71 | 8.20 | 2.19 | 7.86 | --- |
| Nr4a3 | 8.42 | 28.58 | 6.15 | 36.05 | --- |
| Phlda1 | 2.61 | 2.44 | 2.08 | 2.41 | --- |
| Plk2 | 2.43 | 3.57 | 1.98 | 3.76 | --- |
| Ptgs2 | 5.13 | 9.05 | 5.97 | 13.37 | 17.68 |
| Rasl11b | 2.30 | 3.01 | 2.67 | 4.45 | --- |
| Rgs2 | 2.90 | 3.39 | 2.32 | 3.38 | --- |
| Rhob | 1.93 | 2.03 | 1.72 | 2.24 | 8.13 |
| Serpine1 | 5.44 | 2.73 | 1.94 | 2.35 | 8.50 |
| Slc25a25 | 3.34 | 3.42 | 3.01 | 4.46 | 6.94 |
| Thbs1 | 2.06 | 3.06 | 1.86 | 3.24 | 6.52 |
| Tnfaip3 | 2.45 | 3.34 | 3.57 | 5.36 | 6.59 |
| Zfp36 | 5.80 | 9.45 | 7.64 | 15.98 | 23.21 |

**(b) Effects of U0126 on expression of mRNAs upregulated by ET-1 at 30 min.** Cardiomyocytes were unstimulated or exposed to 10 µM U0126 (10 min) then either continued untreated or exposed to 100 nM ET-1 (30 min). Established genes with significantly increased expression at 30 min according to microarray analysis were further studied by SQPCR (SQ) or QPCR (Q). Data are given as fold stimulation relative to unstimulated controls (mean ± SEM, n=4 or 5). Statistical analysis was performed using a one sample t-test with a hypothetical value of zero (i.e. no inhibition).

| **Gene symbol** |  | **% inhibition (mean)** | **SEM** | **Statistical significance** |
| --- | --- | --- | --- | --- |
| **>25% inhibition** | | | | |
| Nr4a3 | SQPCR | 86.6 | 5.1 | <0.0001 |
| Lif | SQPCR | 82.9 | 10.6 | <0.005 |
| Axud1 | QPCR | 78.9 | 6.1 | <0.0005 |
| Egr2 | QPCR | 76.8 | 6.5 | <0.0005 |
| Egr3 | QPCR | 76.6 | 8.7 | <0.001 |
| ATF3 | SQPCR | 73.2 | 8.0 | <0.001 |
| JunB | QPCR | 72.0 | 4.0 | <0.0001 |
| Cited2 | QPCR | 71.5 | 15.2 | <0.001 |
| Arc | SQPCR | 68.3 | 7.4 | <0.001 |
| Il6 | QPCR | 67.9 | 4.3 | <0.0001 |
| Egr1 | QPCR | 67.7 | 5.2 | <0.0005 |
| Ier2 | QPCR | 67.4 | 8.0 | <0.005 |
| RhoB | QPCR | 62.8 | 4.2 | <0.001 |
| Plk2 | SQPCR | 61.9 | 5.5 | <0.005 |
| Zfp36 | QPCR | 61.5 | 6.7 | <0.001 |
| Phlda1 | SQPCR | 61.5 | 8.5 | <0.005 |
| Ereg | QPCR | 61.1 | 10.2 | <0.005 |
| Ptgs2 | QPCR | 57.9 | 7.1 | <0.005 |
| Cyr61 | QPCR | 57.3 | 5.0 | <0.005 |
| Mat2a | QPCR | 57.1 | 20.5 | <0.05 |
| Nr4a2 | SQPCR | 57.0 | 3.7 | <0.0001 |
| Klf2 | QPCR | 56.6 | 3.9 | <0.0001 |
| Pim1 | QPCR | 53.5 | 11.9 | <0.05 |
| Btg2 | SQPCR | 53.0 | 6.3 | <0.005 |
| Dusp5 | QPCR | 51.7 | 9.7 | <0.05 |
| FosB | SQPCR | 51.4 | 7.1 | <0.005 |
| Nfkbiz | QPCR | 51.4 | 4.4 | <0.005 |
| Thbs1 | QPCR | 50.0 | 10.0 | <0.01 |
| Serpine1 | QPCR | 49.1 | 13.8 | <0.05 |
| Klf6 | QPCR | 45.6 | 8.2 | <0.005 |
| Fos | QPCR | 45.6 | 3.9 | <0.0005 |
| Rgs2 | SQPCR | 41.2 | 4.1 | <0.005 |
| Slc25ac25 | QPCR | 38.8 | 10.8 | <0.02 |
| Nr4a1 | SQPCR | 37.8 | 7.0 | <0.05 |
| Nfil3 | QPCR | 33.5 | 8.4 | <0.05 |
| Tnfaip3 | QPCR | 31.4 | 11.3 | <0.05 |
| Jun | QPCR | 26.9 | 7.8 | <0.05 |
|  | | | | |
| **<25% inhibition** | | | | |
| Rasl11b | SQPCR | 23.2 | 25.5 | NS |
| Ier3 | QPCR | 22.2 | 15.5 | NS |
| Ch25h | QPCR | 13.9 | 11.2 | NS |
| C8Orf4 | SQPCR | 12.2 | 16.9 | NS |
|  | | | | |
| **>25% inhibition but not significant** | | | | |
| Myc | SQPCR | 59.2 | 30.7 | NS |
| Has2 | QPCR | 58.4 | 20.6 | NS |
| Klf4 | QPCR | 47.7 | 11.6 | NS (p=0.052) |
| Dusp1 | QPCR | 36.1 | 20.0 | NS |
