# Supplementary material for: Temporal regulation of expression of immediate early and second phase transcripts by endothelin-1 in cardiomyocytes
Source: Genome Biol. 2008 Feb 14;9(2):R32. doi: 10.1186/gb-2008-9-2-r32 (PMC2374717; doi:10.1186/gb-2008-9-2-r32)
Supplement: Additional data file 6 — QPCR data for validation of cycloheximide and polysome clusters. [file gb-2008-9-2-r32-S6.doc]

**Additional data file 6. QPCR validation of cycloheximide and polysome clusters. A,** Total RNA was prepared from untreated cardiomyocytes or cardiomyocytes treated with 20 µM cycloheximide (CX) (10 min), with either no agonist or endothelin (ET) added subsequently for 1 or 2 h. **B**, Cardiomyocytes were untreated (Control) or exposed to ET for 1 h and total or polysome RNA prepared. Gene expression profiles were analysed by Affymetrix Rat Genome 230 2.0 microarrays and by quantitative PCR (QPCR). Expression relative to controls (after normalisation to Gapdh) is shown for the Array data and for QPCR for selected genes. Cluster number relates to Figure 2 and Supplementary Information, Spreadsheet 2 online (cycloheximide data), or Figure 3 and Supplementary Information Spreadsheet 3 online (polysome data). For CX clusters (A), results are the mean expression relative to unstimulated controls for n=3 independent sets of samples. For polysome clusters (B**)**, results are the mean expression relative to total RNA values in unstimulated controls for n=4 independent sets of samples. Each set of samples was prepared from 3 separate cardiomyocyte preparations. **C,** Validation data from A and B were subjected to linear regression analysis to determine the degree of correlation of the microarray data with QPCR analysis. Although the gradient varied according to the individual gene, the correlation coefficient (r) was high for all genes except Hipk. * Affymetrix probe set number for rat expression 230 2.0 arrays (no established gene).

A

| **Gene** | **Cluster** | **Array data** | | | | | | **QPCR** | | | | | |
| --- | --- | --- | --- | --- | --- | --- | --- | --- | --- | --- | --- | --- | --- |
|  |  | **CX**  **1 h** | **ET**  **1 h** | **CX+ET**  **1 h** | **CX**  **2 h** | **ET**  **2 h** | **CX+ET**  **2 h** | **CX**  **1 h** | **ET**  **1 h** | **CX+ET**  **1 h** | **CX**  **2 h** | **ET**  **2 h** | **CX+ET**  **2 h** |
| 1394750* | CX1b | 0.94 | 2.72 | 2.69 | 1.34 | 3.17 | 4.13 | 2.26 | 7.67 | 9.51 | 0.91 | 4.18 | 7.58 |
| Chac | CX2a | 1.31 | 2.65 | 1.74 | 0.80 | 1.23 | 1.29 | 2.01 | 4.37 | 3.02 | 0.97 | 4.11 | 1.85 |
| Dusp2 | CXS3 | 4.17 | 1.44 | 3.96 | 6.12 | 2.46 | 8.04 | 7.59 | 1.92 | 22.88 | 40.12 | 3.09 | 22.31 |
| Dusp5 | CX2c | 1.36 | 4.25 | 3.10 | 1.34 | 4.27 | 1.42 | 2.13 | 3.98 | 4.82 | 2.15 | 4.55 | 2.33 |
| Egr1 | CXS2 | 4.84 | 4.46 | 6.09 | 5.34 | 1.00 | 7.59 | 16.62 | 13.76 | 51.52 | 19.01 | 1.08 | 106.40 |
| Enah | CX1c | 1.39 | 1.46 | 1.74 | 1.67 | 2.44 | 3.60 | 2.22 | 1.25 | 1.82 | 1.75 | 1.94 | 3.12 |
| Ereg | CXS2 | 3.50 | 3.15 | 5.07 | 13.18 | 1.32 | 15.43 | 27.12 | 14.98 | 42.86 | 142.60 | 2.52 | 181.00 |
| Flnc | CX2d | 1.07 | 1.45 | 1.29 | 0.94 | 2.77 | 1.62 | 1.99 | 2.34 | 1.87 | 1.72 | 4.06 | 4.33 |
| Fst | CX2b | 1.82 | 3.61 | 2.38 | 3.71 | 6.00 | 5.33 | 2.60 | 6.35 | 4.93 | 13.56 | 10.71 | 22.06 |
| Has2 | CX2b | 2.03 | 7.54 | 5.41 | 2.86 | 2.39 | 9.79 | 5.06 | 15.81 | 16.38 | 5.68 | 9.69 | 22.10 |
| Hipk3 | CX2a | 1.01 | 1.57 | 1.26 | 1.19 | 1.46 | 1.29 | 1.43 | 0.89 | 1.77 | 0.96 | 1.25 | 1.91 |
| Il1rl1 | CX3a | 1.03 | 2.62 | 1.32 | 1.00 | 4.61 | 0.98 | 1.88 | 13.60 | 3.64 | 1.75 | 15.86 | 2.25 |
| IL6 | CXS1 | 15.03 | 5.58 | 22.07 | 31.09 | 2.37 | 53.37 | 40.02 | 11.40 | 64.64 | 126.50 | 6.82 | 196.90 |
| Irs2 | CX1a | 1.38 | 2.26 | 3.40 | 1.75 | 1.26 | 5.91 | 2.59 | 5.40 | 7.77 | 5.54 | 2.66 | 13.03 |
| Mat2a | CX1a | 1.39 | 2.12 | 2.36 | 1.34 | 1.41 | 2.54 | 1.80 | 3.10 | 3.73 | 2.02 | 2.02 | 3.73 |
| Nfkbiz | CXS1 | 6.28 | 3.05 | 10.75 | 12.03 | 2.29 | 17.74 | 8.54 | 3.75 | 17.08 | 17.61 | 2.17 | 35.64 |
| Nos2 | CX1a | 2.07 | 1.58 | 2.21 | 2.21 | 1.27 | 2.96 | 2.77 | 1.26 | 2.24 | 3.43 | 1.12 | 3.95 |
| RhoB | CXS2 | 2.67 | 2.64 | 4.44 | 3.81 | 1.47 | 6.27 | 8.46 | 4.60 | 22.14 | 12.23 | 1.29 | 51.35 |
| Serpine1 | CX1b | 4.23 | 20.34 | 23.63 | 7.30 | 25.87 | 32.77 | 6.08 | 30.54 | 36.30 | 11.50 | 28.42 | 49.38 |
| Srf | CX1b | 1.14 | 2.10 | 3.12 | 1.01 | 2.07 | 3.31 | 1.45 | 2.90 | 4.66 | 1.00 | 1.39 | 3.13 |
| Tspan5 | CX3b | 1.00 | 1.06 | 0.83 | 0.91 | 3.33 | 1.03 | 1.40 | 1.02 | 2.04 | 1.68 | 6.34 | 1.75 |
| Twist1 | CX3a | 0.91 | 3.31 | 1.26 | 0.86 | 2.88 | 0.93 | 1.80 | 8.60 | 2.09 | 1.28 | 2.47 | 1.55 |

B

|  |  | **Array data** | | | **QPCR data** | | |
| --- | --- | --- | --- | --- | --- | --- | --- |
| **Gene symbol /probe set** | **Cluster** | **Control**  **Polysome** | **ET**  **Total** | **ET**  **Polysome** | **Control**  **Polysome** | **ET**  **Total** | **ET**  **Polysome** |
|  |  |  |  |  |  |  |  |
| 1394750* | PU8 | 0.92 | 2.72 | 1.14 | 0.21 | 6.07 | 0.59 |
| Chac | PU6 | 2.50 | 2.65 | 6.47 | 2.52 | 4.06 | 14.64 |
| Dusp5 | PU5 | 1.40 | 4.25 | 6.41 | 1.42 | 4.17 | 7.79 |
| Egr1 | PU3 | 2.05 | 4.46 | 5.31 | 2.70 | 13.02 | 18.24 |
| Enah | PU2 | 1.18 | 1.56 | 1.32 | 1.44 | 1.72 | 1.34 |
| Ereg | PU5 | 1.03 | 3.13 | 4.87 | 1.15 | 12.51 | 19.76 |
| Flnc | PU7 | 0.36 | 1.62 | 0.50 | 0.15 | 2.43 | 0.36 |
| Fst | PU2 | 1.22 | 3.60 | 4.51 | 1.54 | 5.74 | 10.52 |
| Has2 | PU2 | 0.79 | 7.54 | 6.75 | 0.66 | 15.23 | 17.16 |
| Hipk3 | PU8 | 0.93 | 1.57 | 0.96 | 0.73 | 1.29 | 0.68 |
| IL6 | PU4 | 0.61 | 5.58 | 5.51 | 1.90 | 11.48 | 21.12 |
| Il1rl1 | PU2 | 1.14 | 3.57 | 3.38 | 0.76 | 11.84 | 3.45 |
| Irs2 | PU2 | 0.73 | 2.26 | 1.62 | 0.65 | 4.05 | 2.23 |
| Mat2a | PU4 | 0.62 | 2.12 | 1.49 | 0.33 | 2.13 | 0.87 |
| Nfkbiz | PU2 | 0.71 | 3.05 | 2.80 | 1.02 | 3.35 | 3.34 |
| Nos2 | PU8 | 0.71 | 1.58 | 0.61 | 0.56 | 1.35 | 0.55 |
| RhoB | PU6 | 1.66 | 2.64 | 4.31 | 1.35 | 3.59 | 8.49 |
| Serpine1 | PU2 | 1.24 | 20.61 | 15.64 | 1.03 | 17.89 | 11.49 |
| Srf | PU6 | 1.68 | 2.05 | 3.22 | 1.32 | 2.45 | 6.74 |
| Twist1 | PU6 | 1.60 | 3.20 | 6.34 | 1.65 | 3.84 | 9.48 |

**C**

| **Gene Symbol** | **CX cluster** | **Polysome cluster** | **r** | **Gradient** |
| --- | --- | --- | --- | --- |
| Chac | CX2a | PU6 | 0.96 | 2.30 ± 0.22 |
| Dusp2 | CXS3 | --- | 0.78 | 4.50 ± 1.63 |
| Dusp5 | CX2c | PU5 | 0.95 | 1.06 ± 0.11 |
| Egr1 | CXS2 | PU3 | 0.91 | 3.37 ± 0.51 |
| Enah | CX1c | PU2 | 0.88 | 0.71 ± 0.12 |
| Ereg | CXS2 | PU5 | 0.99 | 12.35 ± 0.61 |
| Flnc | CX2d | PU7 | 0.88 | 1.81 ± 0.33 |
| Fst | CX2b | PU2 | 0.83 | 3.08 ± 0.68 |
| Has2 | CX2b | PU2 | 0.96 | 2.29 ± 0.22 |
| Hipk3 | CX2a | PU8 | 0.33 | 0.53 ± 0.51 |
| Il1rl1 | CX3a | PU2 | 0.85 | 3.60 ± 0.74 |
| Il6 | CXS1 | PU4 | 0.99 | 3.78 ± 0.15 |
| Irs2 | CX1a | PU2 | 0.97 | 2.41 ± 0.19 |
| Mat2a | CX1a | PU4 | 0.92 | 1.71 ± 0.24 |
| Nfkbiz | CXS1 | PU2 | 0.99 | 1.90 ± 0.11 |
| Nos2 | CX1a | PU8 | 0.95 | 1.51 ± 0.16 |
| Rhob | CXS2 | PU6 | 0.88 | 7.77 ± 1.37 |
| Serpine1 | CX1b | PU2 | 0.95 | 1.35 ± 0.15 |
| Srf | CX1b | PU6 | 0.85 | 1.70 ± 0.35 |
| Tspan5 | CX3b | --- | 0.96 | 2.01 ± 0.25 |
| Twist1 | CX3a | PU6 | 0.89 | 1.58 ± 0.26 |
| Unknown (1394750) | CX1b | PU8 | 0.84 | 2.53 ± 0.54 |
