# Supplementary material for: Temporal regulation of expression of immediate early and second phase transcripts by endothelin-1 in cardiomyocytes
Source: Genome Biol. 2008 Feb 14;9(2):R32. doi: 10.1186/gb-2008-9-2-r32 (PMC2374717; doi:10.1186/gb-2008-9-2-r32)
Supplement: Additional data file 7 — Primers used for SQPCR or QPCR. [file gb-2008-9-2-r32-S7.doc]

**Additional data file 7.** Primers used for semiquantitative PCR (SQPCR) or QPCR validation of microarray data. Nucleotide positions in transcripts are shown in parentheses for each primer. mRNA sequences (accession numbers provided) for established genes were obtained from the Rat Genome Database [50].

| **Gene** | **Accession no/Probe set** | **SQPCR** | | | **QPCR** | | |
| --- | --- | --- | --- | --- | --- | --- | --- |
|  |  | **Size (bp)** | **Forward primer** | **Reverse primer** | **Size (bp)** | **Forward primer** | **Reverse primer** |
|  |  |  |  |  |  |  |  |
| Arc | NM_019361 | 447 | CATTCTGCATCAGTGTCCAGG (1709-1729) | GCAGCTTGAGACCTGGTGTC (2136-2155) | --- | --- | --- |
| Atf3 | NM_012912 | 331 | GCTGCCAAGTGTCGAAACAAG (460-480) | CATCGAAGATCGTCGAGTGCT (770-790) | --- | --- | --- |
| Bcr | XM_001079915 | 211 | CGCAGAATTCACAACAGTCCT (893-913) | TCTGCAGCACAGCCATATCCA (1083-1103) | --- | --- | --- |
| Btg2 | NM_017259 | 335 | ACCACCGGTATGAGCCACG (56-74) | ACTTCGTAGGGATCGACCCAC (370-390) | --- | --- | --- |
| Ch25h | NM_001025415 | 317 | CTGCCACAACGTTTCGGAGC (11-30) | GTGACGCTGATGCACTGGG (309-327) | 119 | GAGACGCTACAAGATCCACC (209-228) | GTGACGCTGATGCACTGGG (309-327) |
| C8orf4 (similar) | XM_001072266 | 410 | CTGCAGAGGCTCTTCAGGAA (192-211) | CGAGAGTTCTCAACACCTGC (582-601) | --- | --- | --- |
| Chac1 | XM_342497 | --- | --- | --- | 90 | ACAGGGGCAGCGATAAGATG (620-639) | CTCGAACCTGGTATGCCACA (690-709) |
| Cited2 | NM_053698 | 321 | AGACGGAAGGACTGGAAATG (218-237) | CCCATGAACTGGGAGTTGTT (519-538) | 147 | AGACGGAAGGACTGGAAATG (218-237) | GCTGCTGCTGGTGGTGATG (343-364) |
| Cyr61 | NM_031327 | 269 | ACAATGAGCTCCAGCACCATC (183-203) | CTGACTGAGCTCTGCAGATCC (431-451) | 76 | ACAATGAGCTCCAGCACCATC (183-203) | TGGAGAGTGCCAGCCTGGT (240-258) |
| Dusp1 | NM_053769 | 392 | CCTCCAAGGAGGATATGAAG (486-505) | CAGCATCCTTGATGGAGTCT (858-877) | 139 | CCTCCAAGGAGGATATGAAG (486-505) | GGTGCTACAGGAGCTGCATC (605-624) |
| Dusp2 | NM_001012089 | --- | --- | --- | 142 | TGTGCTTCTTGCGAGGTGGT (452-471) | CCACCCTGGTCATAGATAGG (574-593) |
| Dusp5 | NM_133578 | 218 | TACCATGCATCCAAGTGCGA (748-767) | TCACAGTGAACCAGGACCTT (946-965) | 84 | CGACATTAGCTCCCACTTTC (882-901) | TCACAGTGAACCAGGACCTT (946-965) |
| Egr1 | NM_012551 | 519 | ACCACCCAACATCAGCTCTC (232-251) | AGGCCACTGACTAGGCTGAA (731-750) | 84 | ACAACCCTACGAGCACCTG (550-568) | GGATAACTTGTCTCCACCAG (614-633) |
| Egr2 | NM_053633 | 251 | CCAAGGCCGTAGACAAAATC (354-373) | GTTTCTAGGCGCAGAGATGG (585-604) | 118 | TGATCAGATGAACGGAGTGG (487-506) | GTTTCTAGGCGCAGAGATGG (585-604) |
| Egr3 | NM_017086 | 294 | CCCATTACACTCAGATGGCT (131-150) | GATACATGGCCTCCACGTCT (405-424) | 58 | CCCATTACACTCAGATGGCT (131-150) | TTCTCGTTGGTCAGACCGAT (169-188) |
| Enah | NM_001012150 | --- | --- | --- | 111 | GATTCAAGACCATCAGGTTGTG (255-276) | AGACCATACACCTGTCTAGC (346-365) |
| Ereg | NM_021689 | 274 | ACTGCAGATGTGAAGTGGGC (386-405) | GCTGCACATCCTTGTCCACA (640-659) | 113 | ACTGCAGATGTGAAGTGGGC (386-405) | GAGGAAAACGAGAATCACGG (479-498) |
| Flnc | XM_342653 | --- | --- | --- | 75 | CGTGTCCATCGACAGCAAG (545-563) | AGGATCAGTGTCCAGATCAG (600-619) |
| Fos | NM_022197 | 447 | AGTGGTGAAGACCATGTCAG (464-481) | AATGTTCTTGACCGGCTCCA (892-911) | 132 | AGTAGAGCAGCTATCTCCTG (518-537) | AGTTGATCTGTCTCCGCTTG (630-649) |
| Fst | NM_012561 | --- | --- | --- | 82 | AGTGTGCCATGAAGGAAGCT (881-900) | TCCGAGATGGAGTTGCAAGA (943-962) |
| FosB | XM_001057199 | 241 | CTCCAGCTGTTGACCCTTAT (886-905) | TTAGCTGCAGCCAGCTTGTT (1107-1126) | --- | --- | --- |
| Gapdh | NM_017008 | 453 | ACCACAGTCCATGCCATCAC (1368B1387) | TCCACCACCCTGTTGCTGTA (1801B1820) | 83 | GCTGGCATTGCTCTCAATGACA (1738-1759) | TCCACCACCCTGTTGCTGTA (1801-1820) |
| Has2 | NM_013153 | 304 | CTGGTGAGACAGAAGAGTCCCA (976-997) | ACACTGCTGAGGAAGGAGATCC (1258-1279) | 154 | ATGTACAGGTGTGTGACTCAG (1126-1146) | ACACTGCTGAGGAAGGAGATCC (1258-1279) |
| Hipk3 | NM_031787 | --- | --- | --- | 134 | GGTACTATAGAGCTCCAGAG (1558-1577) | CTGGTCATACTCCAAGGCTC (1672-1691) |
| Ier2 | NM_001009541 | 342 | GACTGGGAAGTGACTTGAGTC (956-978) | GGTCTCCAGCCACTTCAATCA (1277-1297) | 128 | GACTGGGAAGTGACTTGAGTC (1170-1190) | GGTCTCCAGCCACTTCAATCA (1277-1297) |
| Ier3 | NM_212505 | 404 | GCTCTACCCTCGAGTGGTC (199-217) | AGTGTTCTGAGTTAGCGTTGCC (581-602) | 65 | GCTCTACCCTCGAGTGGTC (199-217) | GGATCCTCTTGGCAATGTTG (244-263) |
| Il6 | NM_012589 | ??? | ACCACCCAACATCAGCTCTC (232-251) | ACCACCCAACATCAGCTCTC (232-251) | 134 | CCGGAGAGGAGACTTCACAG (154-173) | GTTGTGCAATGGCAATTCTG (268-287) |
| Il1rl1 | NM_013037 | --- | --- | --- | 84 | GTCTCAAGAGATCGTCTGAAG (418-438) | CGATTCAGGGCTTCTGATAAC (481-501) |
| Irs2 | XM_573948 | --- | --- | --- | 107 | CACCTACGCAAGCATCGACT (3921-3940) | GATTCAGAGTCTTCGACGAG (4008-4027) |
| Jun | NM_021835 | 376 | ATGACTGCAAAGATGGAAACG (859-879) | TATTCTGGCTATGCAGTTCAG (1214-1234) | 140 | GATCATCCAGTCCAGCAATG (1095-1114) | TATTCTGGCTATGCAGTTCAG (1214-1234) |
| Junb | NM_021836 | 225 | ATCACGACGACTCATACGCA (299-318) | TGGAGGCTAGCTTCAGAGAT (504-523) | 148 | AAACCCACCTTAGCGCTCAA (376-395) | TGGAGGCTAGCTTCAGAGAT (504-523) |
| Klf2 | NM_001007684 | 173 | ACTTGCAGCTACACCAACTG (805-824) | CTGTGACCCGTGTGCTTG (960-977) | 173 | ACTTGCAGCTACACCAACTG (805-824) | CTGTGACCCGTGTGCTTG (960-977) |
| Klf4 | NM_053713 | 231 | TGCCAGACCAGATGCAGTC (1140-1158) | CACAGTGGTAAGGTTTCTCG (1351-1370) | 175 | TCAAGAGCTCATGCCACCGG (1180-1199) | CTCGCCTGTGTGAGTTCGCA (1335-1354) |
| Klf6 | NM_031642 | 287 | CTGAGTTCCTCGGTCATTTC (587-606) | TCCCAAGAGCATCTGTAAGG (854-873) | 126 | CCTTACAGATGCTCTTGGGA (854-873) | GGAGAAACACCTGTCACAGT (960-979) |
| Lif | NM_008501 (mouse) | 478 | ATGAAGGTCTTGGCCACAG (224-242) | ACTTGTTGCACAGACGGCAA (682-701) | --- | --- | --- |
| Mat2a | NM_134351 | 505 | GTGCAGGAGATCAGGGTTTGA (469-489) | CGAGCAGCATAAGCAGCTGAA (953-973) | 174 | GTGCAGGAGATCAGGGTTTGA (469-489) | TATACTGCACAGTCACTTGAGT (621-642) |
| Myc | NM_012603 | 173 | ACGGCCTTCTCTTCTTCCTC (1221-1240) | GGTTGCCTCTTTTCCACAGA (1374-1393) | --- | --- | --- |
| Nfil3 | NM_053727 | --- | --- | --- | 122 | TGGGTCACAGCCATCCGTT (90-108) | GCTTCAGCTTCTCGAATCCA (192-211) |
| Nfkbiz | XM_221537 | 282 | CGCTCAACCTCGCTTACTTC (190-209) | GCAGCTCTTTCACCGAGTTC (452-471) | 87 | CGCAAGTTGAGCCCCATATG (385-404) | GCAGCTCTTTCACCGAGTTC (452-471) |
| Nos2 | NM_012611 | --- | --- | --- | 96 | CTATTCCCAGCCCAACAACA (286-305) | CTGGAACATTCTGTGCAGTC (362-381) |
| Nr4a1 | NM_024388 | 306 | CTTCTTCAAGCGCACAGTAC (974-993) | ACCAGTTCCTGGAACTTGGA (1260-1279) | --- | --- | --- |
| Nr4a2 | NM_024388 | 517 | TTTCTTTAAGCGCACGGTGC (966-985) | TGGGTTGGACCTGTATGCTA (1463-1482) | --- | --- | --- |
| Nr4a3 | NM_031628 | 293 | GCAGCAGTTCTACAACCTTC (2007-2026) | GGATATCAAGGTTCAGGCTC (2280-2299) | --- | --- | --- |
| Phlda1 | NM_017180 | 241 | ACCCTCATCCACACCAACTC (1109-1128) | ATCCGTAGGGTGATGTCCAA (1330-1349) | --- | --- | --- |
| Plk2 | NM_031821 | 406 | GGATCTCAAGCTAGGGAACT (734-753) | AGTGAAGCCCTGCAGAAAGA (1120-1139) | --- | --- | --- |
| Ptgs2 | NM_017232 | 283 | CACAAATATGATGTTCGCATTC (661-682) | CACAGCGAACCGCAGGTG (926-943) | 106 | GAAATATCAGGTCATCGGTGGAG (838-860) | CACAGCGAACCGCAGGTG (926-943) |
| Rasl11b | NM_001002830 | 264 | CAAGACCGCTTTGGTGGTC (292-310) | TGGAGCTGGCTAATGAGTTC (536-555) | --- | --- | --- |
| Rgs2 | NM_053453 | 354 | GAAGCGGACACTCTTAAAAGA (112-132) | TCTATGTTTATCTCTTTGGGAGC (443-465) | --- | --- | --- |
| Rhob | NM_022542 | 367 | GGACACCGACGTCATCCTTA (507-526) | TCATAGCACCTTGCAGCAGT (854-873) | 133 | CTATGACTACCTCGAGTGCT (741-760) | TCATAGCACCTTGCAGCAGT (854-873) |
| Serpine1 | NM_012620 | 159 | CTCTCTGTAGCACAAGCACT (1130-1149) | TGTTGGATTGTGCCGAACCA (1269-1288) | 159 | CTCTCTGTAGCACAAGCACT (1130-1149) | TGTTGGATTGTGCCGAACCA (1269-1288) |
| Slc25a25 | NM_145677 | 311 | CCTTGATGGGCAACTGGACT (286-305) | CATCGAAGATCGTCGAGTGCT (576-596) | 58 | CCCTGTCACCTACATGGATAAG (131-150) | CATCGAAGATCGTCGAGTGCT (169-188) |
| Srf | XM_576514 | --- | --- | --- | 131 | ACCATCCACCATGCAGGTGT (1641-1660) | TCACAGCCATCTGGTGAAGC (1752-1771) |
| Thbs1 | NM_001013062 | 666 | TGGAAGCAACCGCATTCCAG (83-102) | ACGTTGGTTGAACTGGAGCA (729-748) | 91 | TTCAGGGGGTGCTGCAGAATG (658-678) | ACGTTGGTTGAACTGGAGCA (729-748) |
| Tnfaip3 | XM_001060980 | 376 | TGACGGCTGATCGGCCACT (194-212) | CAGTTTCCATCACCGTTGGT (550-569) | 143 | TGACGGCTGATCGGCCACT (194-212) | GGTTCTCTCTCGTATCTTCAC (316-336) |
| Tspan5 | NM_001004090 | --- | --- | --- | 107 | AACTGCACAGACTCCAATGC (510-529) | GCATCATAGCCACACTGAGT (597-616) |
| Twist1 | NM_053530 | --- | --- | --- | 67 | CGGAGACCTAGATGTCATTG (821-840) | TCCAGCTCCAGAGTCTCTAG (868-887) |
| Unknown | 1394750_AT | --- | --- | --- | 80 | AGAGTCCTTGCTAAGTCTGC (24-43) | TTTTGTGGATGGAACCGACG (84-103) |
| Zfp36 | NM_133290 | 292 | CATCTACGAGAGCCTTATGTC (58-78) | ACAGAGCTCAGTCTTGTATCG (329-349) | 107 | CATCTACGAGAGCCTTATGTC (58-78) | ATGGGATGGAGTCCGATGAG (145-164) |
